# Methods of Stock-Keeping

**Published:** 1905-10-28

**Authors:** 


### The Uniform System of Accounts.

### METHODS OF STOCK-KEEPING.

That a periodic stock-taking is needed in every institution is understood, but unfortunately there is as yet no such universal method of stock-keeping as has been introduced into hospital book-keeping by the uniform system of accounts. From many institutions comes the grumble that necessary replenishments cannot be obtained when wanted, because "matron"—or whoever has charge of the stores—does not remember when the last supplies were furnished. In some cases renewals are not even asked for as old things are worn out, because one can more or less rub along for a time with less than the original stock. Meanwhile pieces of lint or towels are used for dusters, and so forth until the whole supplies are in confusion. The first thing is, obviously, to decide how many of each of the articles required a certain ward should be supplied with, and then to keep the supplies always up to the standard. A very simple method, which is in vogue in some institutions, keeps before the eyes of the sister or whoever is in charge of the particular department, what this supply should be. Thus, suppose it is decided that the number of sheets needed for Ward A is 200, let each of these sheets be marked "A 200." This shows at once to what ward the sheet belongs, and how many there ought to be. In time the sheet will wear out. It should then be taken to the store-room and exchanged for a new one similarly marked. Thus there would never at any time be less than the required 200 sheets belonging to Ward A. To use a torn sheet for any other purpose without returning it to the store-room should be set down as a fault in whoever was guilty of it. The matron would doubtless use up the best parts of it for some other purpose, but it should be the rule that it should go first through her hands. It should be no excuse for the deficiency in the quantity of any article that a nurse, when it was worn, had used it for some other than its original purpose. Even the broken crockery of the week should be in evidence when new items were asked for. This would soon show in what departments of the institution there was waste or carelessness, and give the matron data for inquiry or reproof when these were needed. Rags, scrap-iron, and the like can be sold when no further use can be found for them, and the sums, comparatively trifling as they might be, derived from such a source would at least equal a modest subscription to the institution funds, but more helpful than that would be the check on carelessness and extravagance given by the knowledge that everything had to be accounted for to those in authority. There is, of course, one thing in which it is impossible to insist on this return of the used articles—viz., lint and other dressings, in which there is undeniably often great waste. But the habit of carefulness inculcated with other things might reasonably be expected to check the habit of extravagance with these, and lead to a general rule of economy. Even if it did not, the fact that no check can be put to the employment of some things is no excuse for carelessness in the use of others. We know that in some institutions the management of the stores is as accurate and careful as possible, but it would be folly to pretend that it is always perfect. It is to recall to the minds of our readers the importance of a part of their work which they may regard as uninteresting that we have dwelt upon the matter.

